# Supplementary material for: Tumor microenvironment-induced FOXM1 regulates ovarian cancer stemness
Source: Cell Death Dis. 2024 May 28;15(5):370. doi: 10.1038/s41419-024-06767-7 (PMC11133450; doi:10.1038/s41419-024-06767-7)

Supplementary Figure 1.

(A) Phase-contrast pictures of normal omental fibroblasts (NOF) and hman primary mesothelial cells (HPMC) derived from a human omental specimen. Scale bar: 400 μm. (B) Immunofluorescence for WT1 (in green) and αSMA (in red) on NOF and HPMC derived from a human omental specimen. Nuclei were counterstained with DAPI (blue). Scale bar: 50 μm. (C) Phase-contrast and fluorescence pictures of the organotypic TME alone or co-cultured with CMFDA-stained bulk cells and OCSC from OC3 sample. Scale bar: 400 μm.

Supplementary Figure 2.

(A) Gene Set Enrichment Analysis (GSEA) of FOXM1 transcription factor network on the ranked expression data matrix of OCSC after co-culture with TME. NES, normalized enrichment score. FDR, false discovery rate. (B) Immunofluorescence for FOXM1 (in red) on CMFDA-labelled OCSC from OC1 sample (in green) co-cultured with TME, or on OC1 OCSC spheres only (lower panel). Nuclei were counterstained with DAPI (blue). Scale bar: 50 μm. (C) Violin plot showing the expression level of the *FOXM1* gene in FOXM1 positive cells from two patients (OC1 in blue and OC4 in orange) divided by sample type (x axis).

Supplementary Figure 3.

Gene Set Enrichment Analysis (GSEA) of HALLMARK_MITOTIC_SPINDLE, HALLMARK_G2M_CHECKPOINT and HALLMARK_E2F_TARGETS gene sets on ranked expression data matrix of OCSC after co-culture with TME and on the ranked expression data matrix of TCGA-OC cohort correlating with FOXM1 expression profile (High → Low), showing a significant enrichment for all these signatures in both datasets.

Supplementary Figure 4.

(A) Cell viability of OC7 and OC8 primary samples measured after 72h of treatment with vehicle (DMSO) or 3 different doses FOXM1 inhibitor Thiostrepton. (B) Sphere formation efficiency in OC7 and OC8 primary samples treated or not with Thiostrepton 10 μM. Comparisons between experimental groups were done with two-sided Student's t-test; $*p < 0.05$, $***p < 0.005$. (C) mRNA expression of *FOXM1* was analyzed by qRT-PCR in a panel of ovarian cancer cell lines (bulk OC or OCSC) cultured with TME for 48h. Data are represented as a relative mRNA expression ($2^{-\Delta\Delta Ct}$) of bulk (in black) and OCSC (in grey) co-cultured with TME for 48 hours (T=48h) compared to cells grown in absence of TME (T=0, dashed line). Comparisons between experimental groups were done with two-sided Student's t-test; $***p < 0.005$. (D) Cell lysates from TYK-nu cells control (Scramble)

and silenced for FOXM1 with 2 different shRNAs (shFOXM1-C and E) were immunoblotted for FOXM1 and beta-tubulin was used as loading control. (E) Growth curves of TYK-nu Scramble or knocked down for FOXM1 expression with shFOXM1-C and E. Proliferation was evaluated by crystal violet staining. Data are represented as mean ± SD from one representative experiment (n=3). (F) Nude mice were transplanted subcutaneously with decreasing numbers of either TYK-nu Scramble or TYK-nu shFOXM1-C cells, and analyzed for tumor take 21 days after injection.

Supplementary Figure 5.

(A) Conditioned medium (CM) from 2 different *in vitro* co-cultures of TME was used to treat TYK-nu OCSC, followed by immunoblotting of cell lysates for FOXM1. Vinculin was used as loading control. (B, C) mRNA expression of *FOXM1* was analyzed by qRT-PCR in TYK-nu OCSC (B) or in primary OCSC from 4 primary samples (C) cultured on collagen or fibronectin for 24h. Data are represented as a relative mRNA expression ($2^{-\Delta\Delta Ct}$), compared to OCSC only (dashed line). Comparisons between experimental groups were done with two-sided Student's t-test; ***$p < 0.005$. (D) Gene Set Enrichment Analysis (GSEA) of CORDENONSI_YAP_CONSERVED_SIGNATURE dataset on ranked expression data matrix of OCSC after co-culture with TME and on the ranked expression data matrix of TCGA-OC cohort correlating with FOXM1 expression profile (High → Low), showing a significant enrichment for YAP signature in both datasets. NES, normalized enrichment score. FDR, false discovery rate (aka q-value). (E) Cell lysates from TME cultured or not with TYK-nu OCSC and treated for 24h with vehicle (DMSO) or Verteporfin 3 μM (VP) were immunoblotted for FOXM1 and YAP, while vinculin was used as loading control. The panel shows a single blot, intervening lanes were removed for clarity reasons. (F) Cell lysates from TME cultured or not with TYK-nu OCSC and treated for 24h with vehicle (DMSO) or Defactinib 1 μM (DEF) were immunoblotted for FOXM1, pFAK and FAK, while vinculin was used as loading control. (G) Cytosolic and nuclear lysates from TYK-nu cells treated for 24h with vehicle (DMSO) or Defactinib 1 μM (DEF) were immunoblotted for YAP; beta-tubulin was used as loading control for the cytosolic fractions, while Lamin A/C was used for nuclear fractions.

Supplementary Figure 1

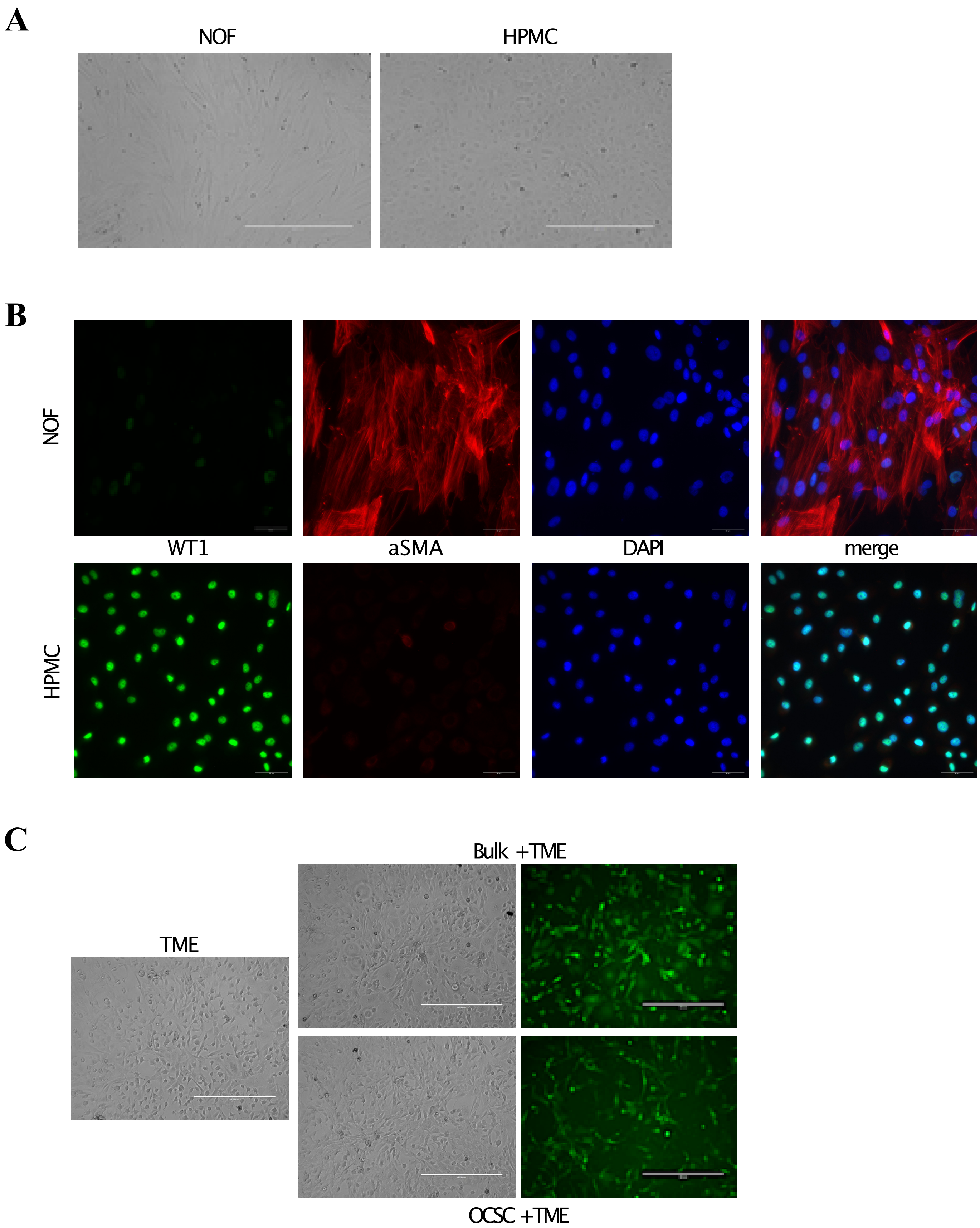

Supplementary Figure 2

A

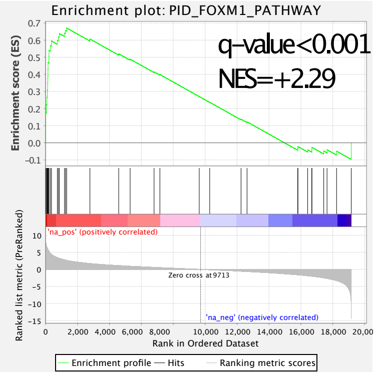

B

FOXM1/CMFDA/DAPI

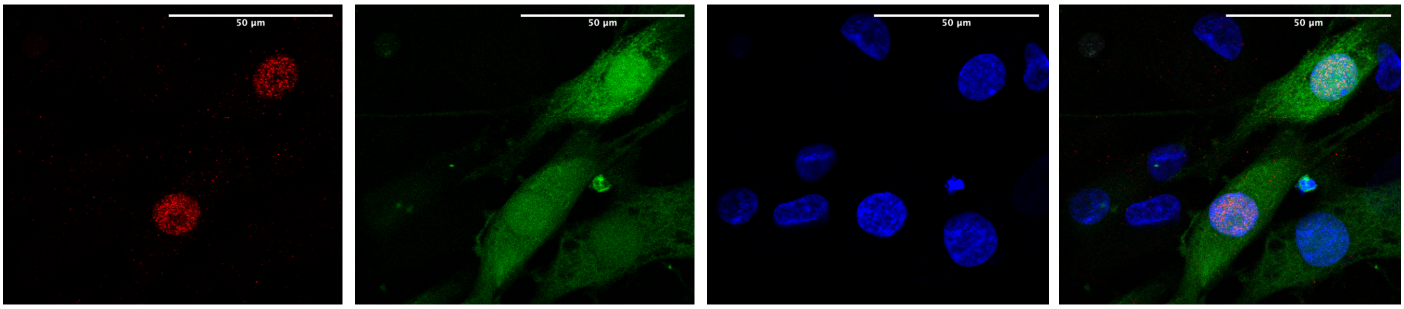

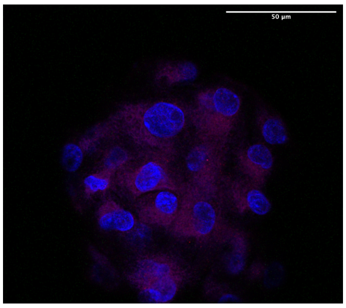

FOXM1/DAPI

C

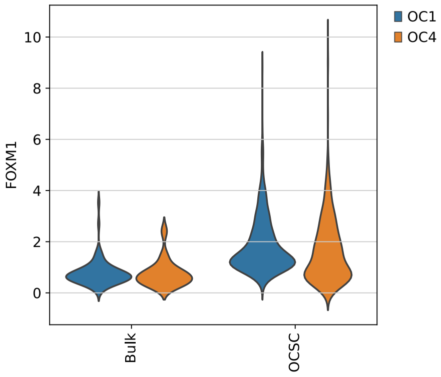

Supplementary Figure 3

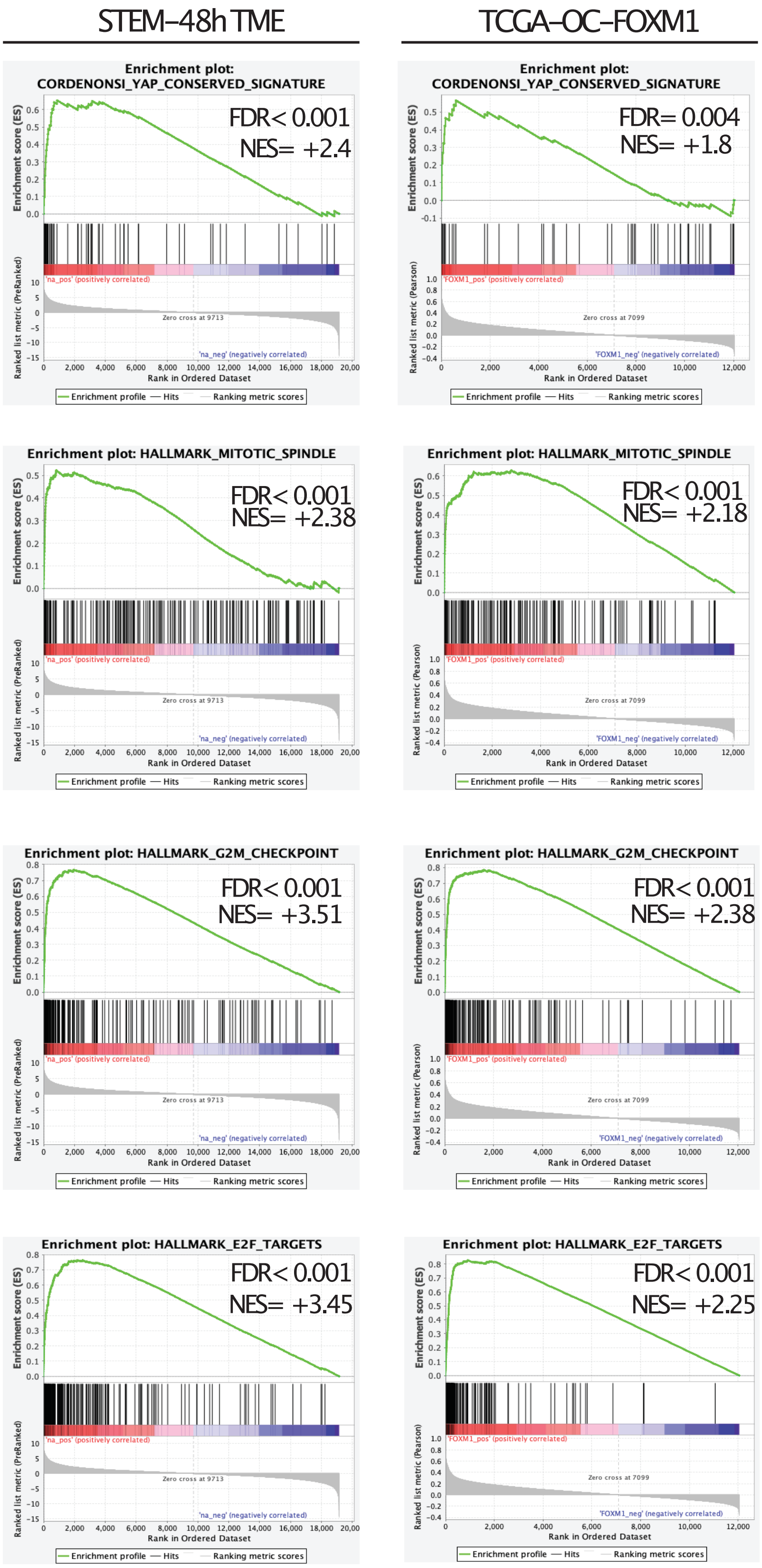

Supplementary Figure 4

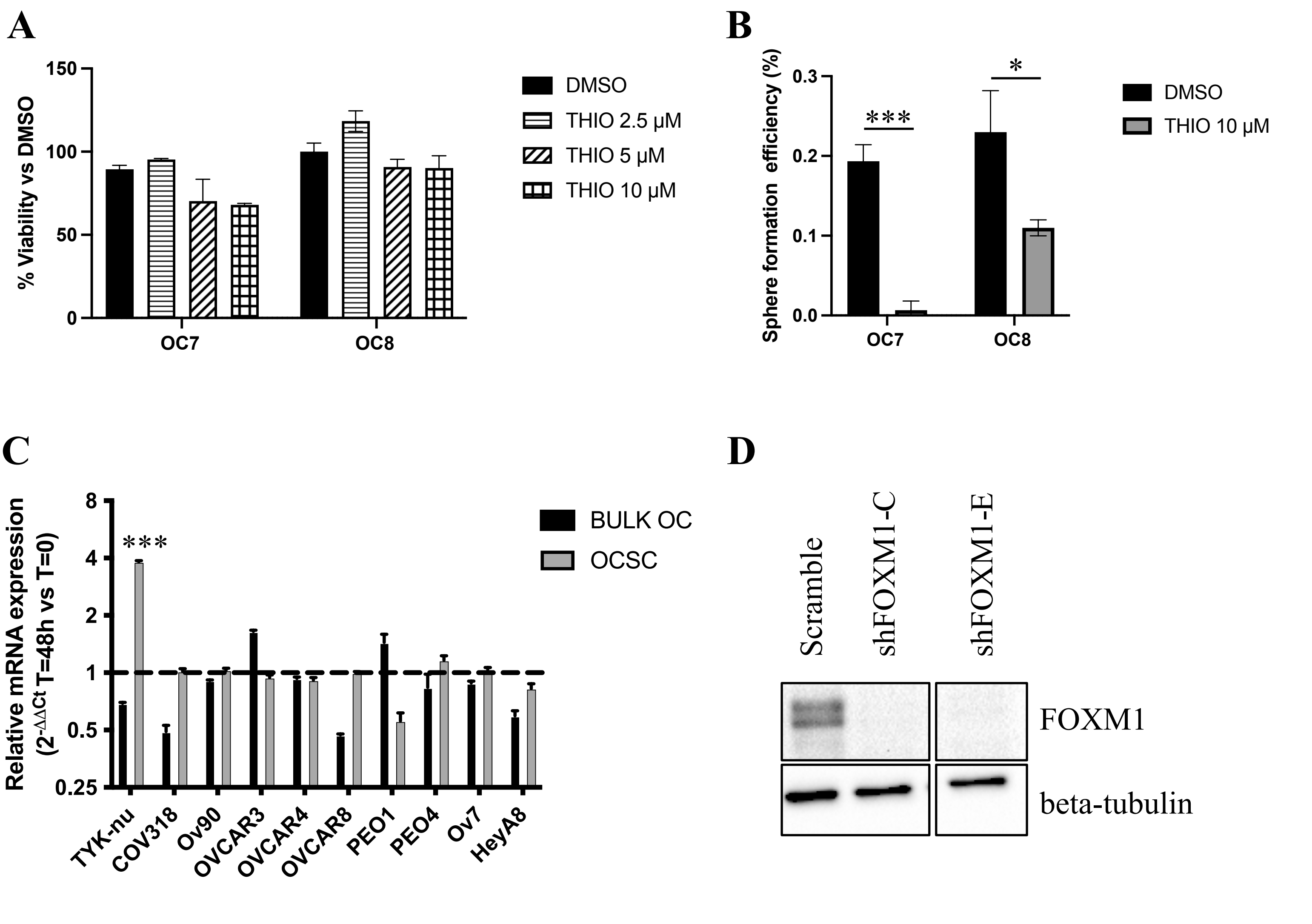

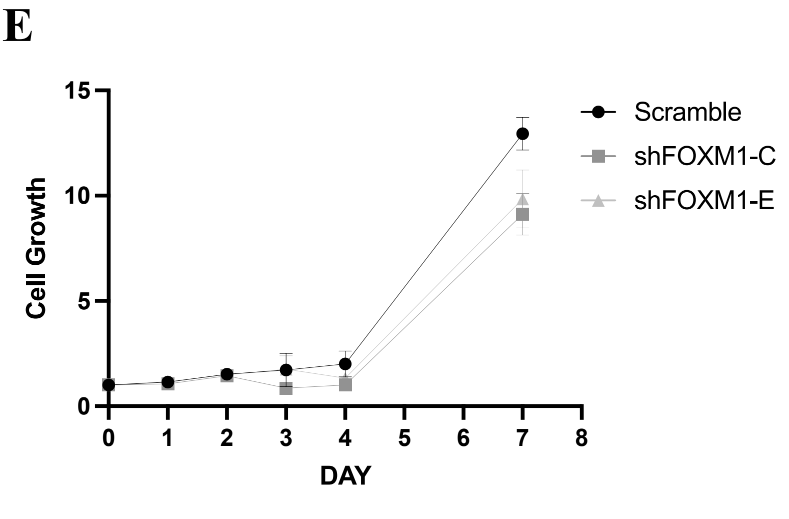

F

Group	N. of transplanted cells	Tumor take
Scramble	5*10^6	7/7
	1*10^6	7/7
	2.5*10^5	5/7
	1*10^5	6/7
	1*10^4	2/7
shFOXM1	5*10^6	7/7
	1*10^6	3/7
	2.5*10^5	5/7
	1*10^5	6/7
	1*10^4	0/7

Supplementary Figure 5

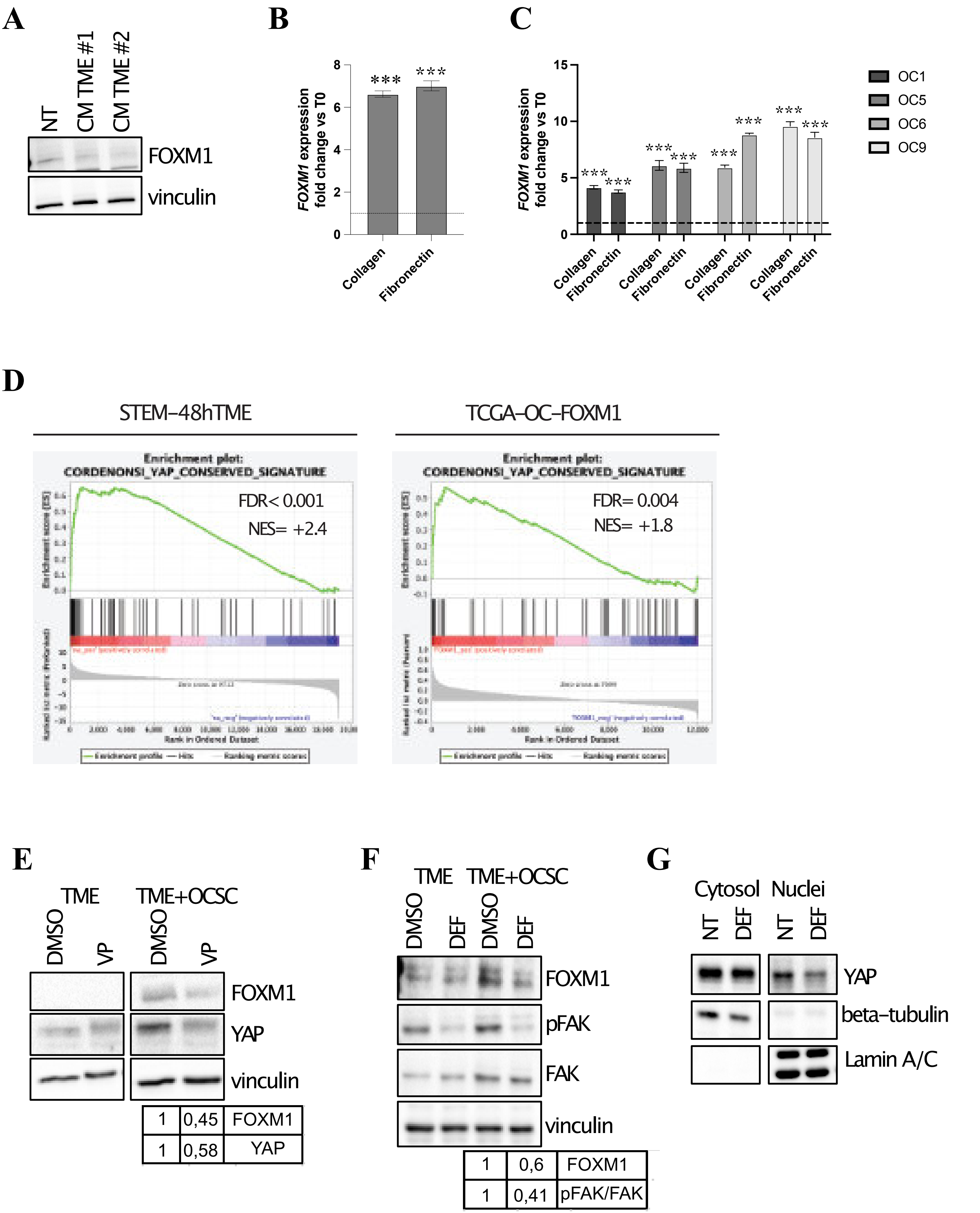

Supplement: Supplementary file 1 — Supplementary figures 1-5 + legends [file 41419_2024_6767_MOESM1_ESM.pdf]
