# Supplementary material for: Tumor microenvironment-induced FOXM1 regulates ovarian cancer stemness
Source: Cell Death Dis. 2024 May 28;15(5):370. doi: 10.1038/s41419-024-06767-7 (PMC11133450; doi:10.1038/s41419-024-06767-7)

Figure 4A

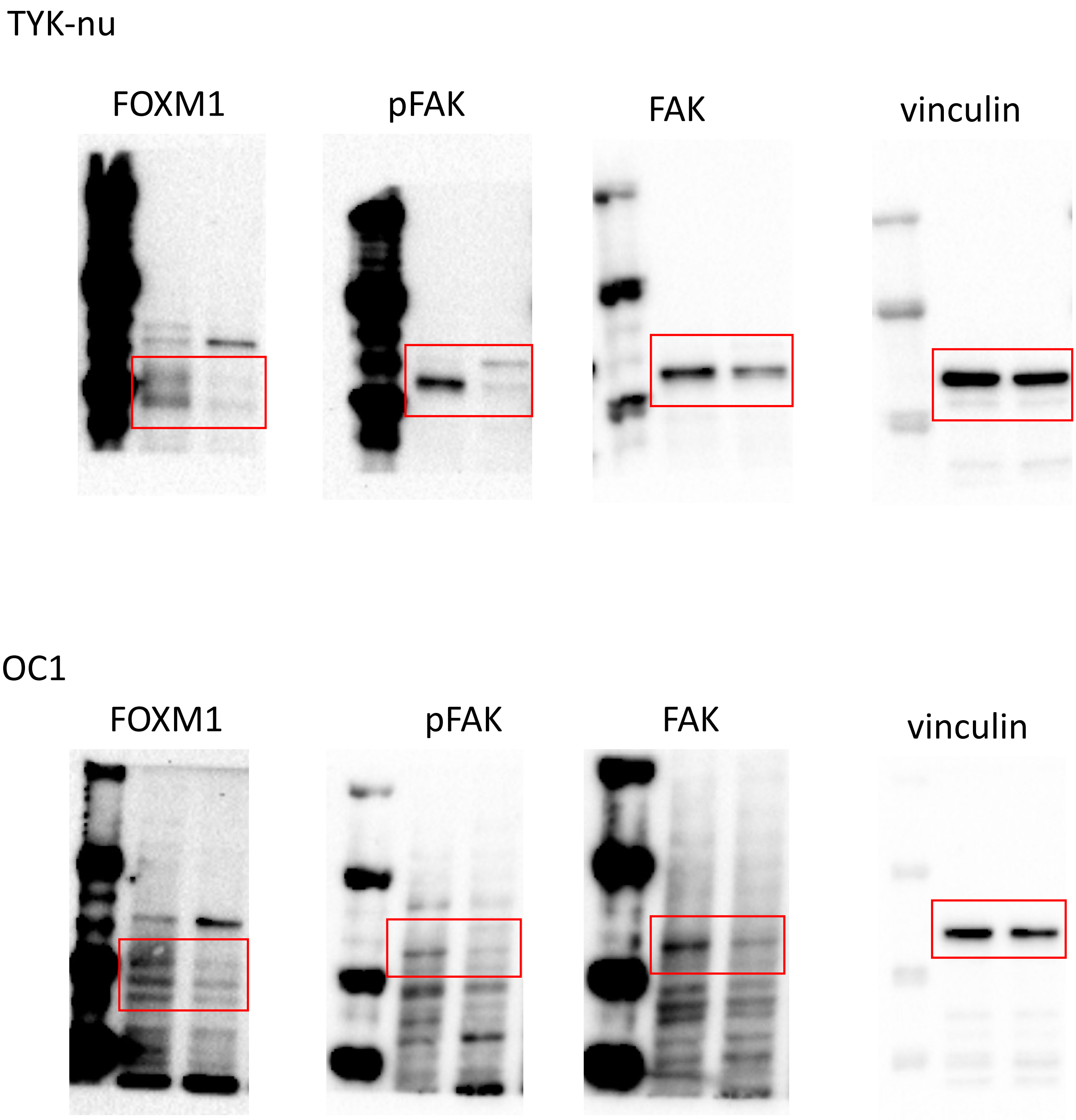

Supplementary Figure 4D

FOXM1

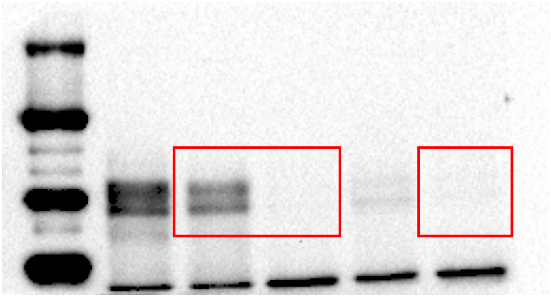

beta-tubulin

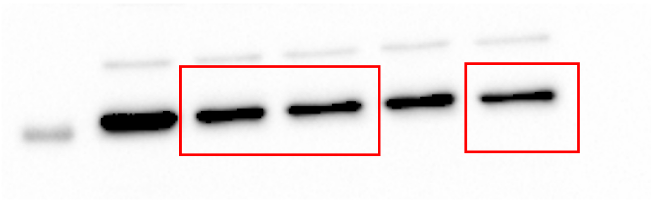

Supplementary Figure 5

A

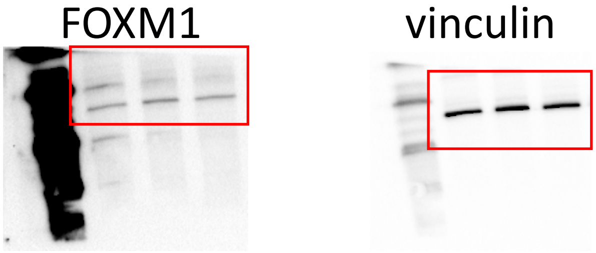

E

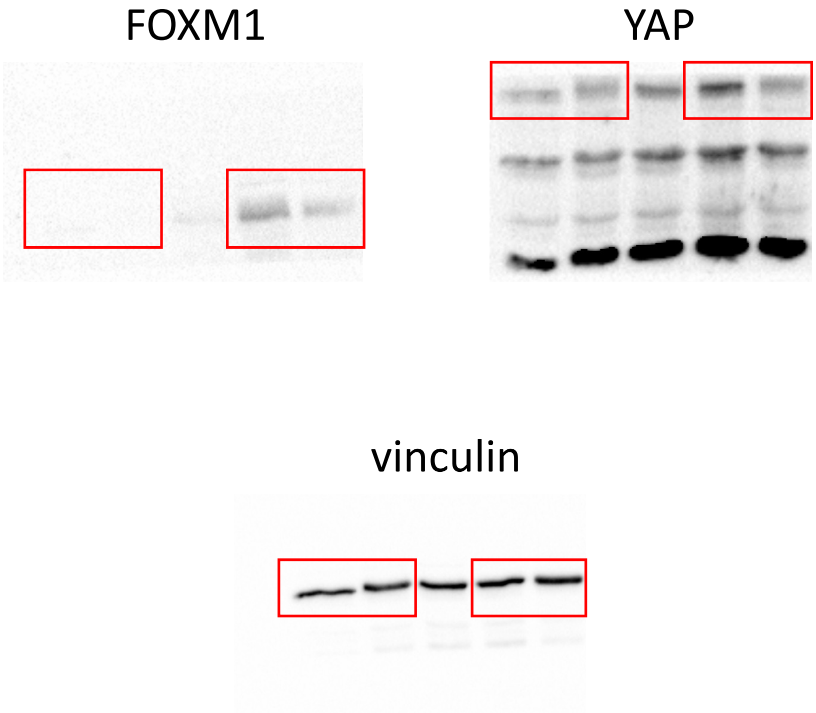

Supplementary Figure 5

F

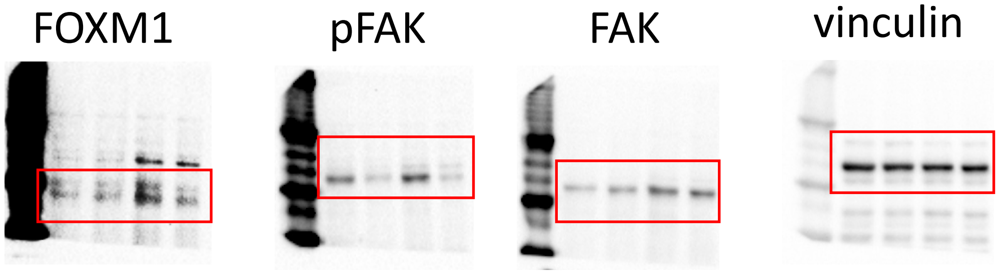

G

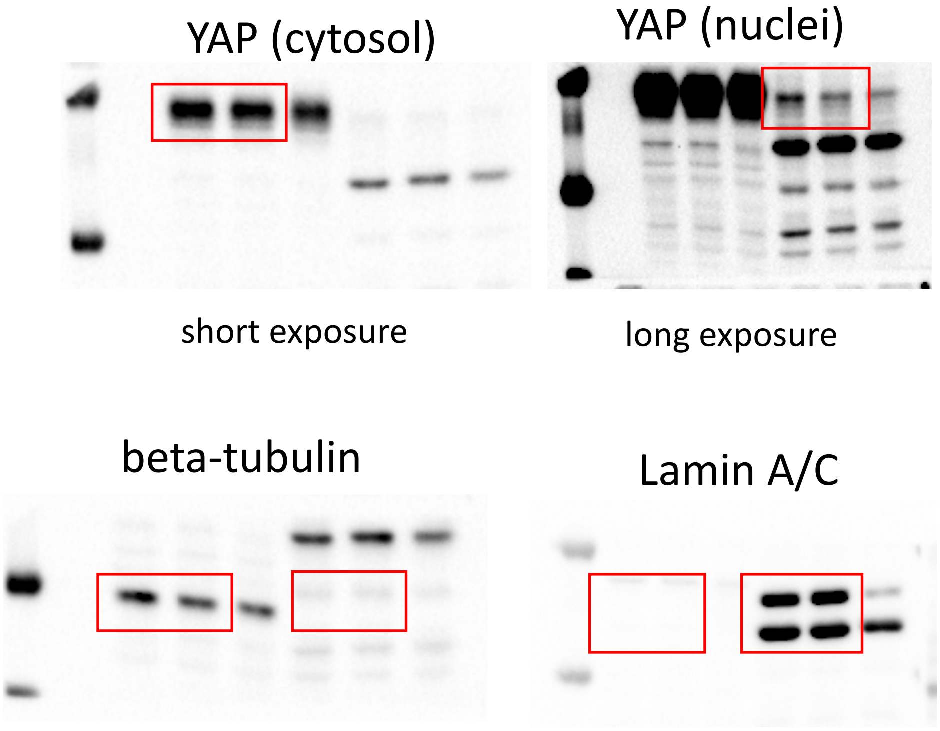

Supplement: Supplementary file 2 — Uncropped western blots [file 41419_2024_6767_MOESM2_ESM.pdf]
